# Monte Carlo modeling of the formation and organization of ion channel clustering

**DOI:** 10.1371/journal.pcbi.1014791

**Published:** 2026-09-17

**Authors:** Nicolae Moise, Seth H. Weinberg

**Affiliations:** 1 Department of Biomedical Engineering, The Ohio State University, Columbus, Ohio, United States of America; 2 Davis Heart and Lung Research Institute, The Ohio State University Wexner Medical Center, Columbus, Ohio, United States of America; Lehigh University, UNITED STATES OF AMERICA

## Abstract

The spatial organization of ion channels on cell membranes critically influences many key physiological processes, such as cardiac and neuronal excitability and cellular signaling, yet the mechanisms governing channel clustering remain poorly understood. In this study, we present a stochastic computational framework that models the dynamic organization of ion channels through Monte Carlo simulations incorporating membrane insertion, removal, channel-channel interactions, and diffusion processes. Our model reveals several fundamental principles of membrane domain formation. In single-channel systems, we demonstrate a biphasic relationship between interaction energy and cluster size, with optimal clustering occurring at intermediate interaction strengths, suggesting that excessively strong interactions can impede cluster growth by restricting channel mobility. In two-channel systems, we find that the interplay between homotypic and heterotypic interactions determines whether channels form mixed or segregated clusters, with asymmetric clustering behaviors emerging when homotypic interaction strengths differ between channel types. Simulations of three-channel systems demonstrate emergent organizational principles leading to hierarchical clustering patterns and specialized domain formation. These findings generate testable predictions about how channel density, trafficking dynamics, and interaction energies collectively alter ion channel spatial organization, in the setting of both physiological function and pathophysiological conditions.

## Author summary

The organization of ion channels into functional clusters on cell membranes remains poorly understood. This study presents a novel computational framework that simulates dynamic channel clustering by integrating trafficking, diffusion, and channel-channel interactions. The model reveals counterintuitive relationships, including how intermediate rather than strong interactions optimize cluster formation, and demonstrates how competition between different interaction types creates complex spatial patterns in multi-channel systems. This framework enables quantitative predictions on the relationship between channel density, trafficking rates, and interaction energies collectively shape membrane organization. These insights have important implications for understanding disorders in which disrupted channel organization contributes to disease pathophysiology, offering a foundation for predicting how mutations or cellular changes affect membrane organization.

## Introduction

Ion channels are critical regulators of cellular signaling, in excitable cells, such as neurons and cardiac myocytes, and non-excitable cells, including immune cells, fibroblasts, cancer cells, pancreatic β-cells, pituitary cells, vascular and pulmonary endothelial cells, amongst nearly all other cell types [1–7]. As such, ion channels are ubiquitous drivers of physiological processes and the spatial organization of ion channels on cellular membranes plays a critical role in many key physiological processes such as electrical excitation, synaptic transmission, muscle contraction, proliferation, cytokine and hormone secretion, etc.

A single ion channel type rarely if ever functions in isolation. Rather, multiple ion channel and transporter/exchanger proteins work in concert to perform a specific physiological function, highlighting the necessity for channel co-regulation and coordination. Ion channels typically form clusters of varying density, and indeed emerging research, including our own, highlights that clusters of multiple different channel types co-localize at distinct locations within cells, often specialized ultrastructural niches [8–22]. These clusters can include single channel types (homotypic clusters) or multiple channel types (heterotypic clusters), with emerging evidence suggesting that the precise organization of these clusters significantly influences cellular physiology under both normal and pathological conditions [23–26].

Despite the importance of ion channel clustering, the fundamental mechanisms that govern their formation, maintenance, and regulation remain poorly understood. While experimental approaches using super-resolution microscopy have revealed increasingly detailed snapshots of channel organization [21,27], these techniques are limited in their ability to capture the dynamic processes that lead to cluster formation. Additionally, the multitude of potential interactions, including channel-channel, channel-lipid, and channel-cytoskeleton interactions, creates a complex system that is difficult to dissect experimentally. The formation and maintenance of these multi-channel clusters is complex, involving co-regulated protein trafficking, recycling, scaffolding, diffusion, and protein binding. While many models of ion channel function (e.g., gating kinetics) exist [28–30], there are very few to consider channel clustering structure.

Computational modeling offers a powerful complementary approach to understand the principles governing ion channel organization. Prior models of membrane-protein organization have focused on cytoskeletal regulation of clustering based on principles of thermodynamical and/or mechanical equilibrium [31,32], for example, illustrating how active actin dynamics regulate clustering of lipid-anchored proteins at the membrane [33]. These studies thus consider an “intracellular-centric” perspective on protein clustering regulation. In contrast, “membrane-centric” modeling studies have often focused on membrane lipid-protein interactions, for example, all-atom Molecular Dynamics simulations demonstrating the role of lipid structure in supporting protein clustering [34]. Yet, these simulations are limited in the spatial and temporal scope due to computational constraints. By simplifying these membrane-protein and protein-protein interactions to thermodynamical energy constraints and intracellular regulation to probabilistic protein trafficking, our proposed model can simulate a wide range of conditions and scales in both space and time.

To address these gaps, in this study, we develop a novel stochastic computational framework that simulates the dynamic organization of ion channels on the cell membrane. The Monte Carlo approach incorporates four key processes that influence channel spatial organization: membrane removal, membrane insertion, channel-channel interactions, and diffusion. Critically, our model allows for the systematic investigation of how channel clustering is influenced by factors such as channel density, trafficking rates, and interaction energies between similar or different channel types.

We previously illustrated the clustering model framework as a single proof-of-concept to predict altered multi-channel spatial organization in the setting of a loss of expression channel mutation [23]. In this study, we perform a systematic study of the clustering model to explore how the balance between trafficking dynamics and interaction energies shapes cluster formation for channel types, revealing non-intuitive relationships between these parameters and clustering outcomes. We extend the model to investigate systems with multiple channel types, illuminating how competition between homotypic and heterotypic interactions leads to complex spatial patterning. Finally, we characterize emergent properties in three-channel systems that demonstrate how selective affinities between different channel types could organize the membrane into functionally specialized domains. By providing a quantitative framework for understanding ion channel organization, our model generates testable predictions about how alterations in channel properties or cellular conditions might affect membrane organization and, consequently, cellular function. These insights have potential implications for understanding channelopathies and other disorders in which disrupted channel organization contributes to disease pathophysiology.

## Materials and methods

### Model formulation

In the channel clustering model, individual ion channels are represented by pixels on a 2D grid lattice, with dimensions $N_{x}$ x $N_{y}$ and pixel size Δx x Δx (where we have assumed equal grid spacing). In this study, we define $N_{x}=N_{y}=100$ and Δx=10nm, based on realistic ion channel size [25], such that we simulate a membrane patch of 1 μm x 1 μm. For all conditions investigated, channel dynamics are described by four stochastic processes: channel insertion, channel removal, channel-channel interactions, and channel diffusion. Simulations are performed by a Monte Carlo method, described as follows:

### Channel seeding and iteration

First, for channel type *i*, for $i=1,2,...,N_{chan}$, where $N_{chan}$ is the number of channel types in the membrane, channels are randomly seeded on the membrane lattice, with probability $p_{seed,i}$. Then, $N_{time}$ iterations are performed that evolve channel location and insertion to/removal from the membrane: For each iteration *n*, for $n=1,2,...,N_{time}$, a random pixel (*x*,*y*) is selected.

#### Channel insertion and removal.

If the pixel is ‘unoccupied,’ i.e., there is no channel present, then channel type *i* is inserted into the membrane with probability $p_{insert,i}$. If the selected pixel is ‘occupied,’ i.e., there is a channel present, then the channel is removed from the membrane with probability $p_{remove,i}$.

For the case of individual channel insertion/removal, we can define the relationship between the expected number of type *i* channels on the (*n* + 1)th iteration $N_{i}(n+1)$ and the number of free membrane pixels $N_{mem}(n)$ and type *i* channels on the *n*th iteration $N_{i}(n)$:


$$\begin{array}{c} N_{i}(n+1)=p_{insert,i}N_{mem}(n)+(1-p_{remove,i})N_{i}(n). \end{array}$$ (1)

Accounting for (i) the number of free membrane pixels $N_{mem}(n)=N_{x}N_{y}-\sum_{i=1}^{N_{chan}}N_{i}(n)$, (ii) at steady-state $N_{i}^{*}\approx N_{i}(n)\approx N_{i}(n+1)$, and (iii) defining the steady-state membrane fraction of channel type *i* as $f_{chan,i}=N_{i}^{*}/(N_{x}N_{y})$, the general relationships for the steady-state membrane fractions $f_{chan,i}$ are given by:


$$\begin{array}{c} (p_{insert,i}+p_{remove,i})f_{chan,i}+p_{insert,i}\sum_{j=1,j=\;\;/i}^{N_{chan}}f_{chan,j}=p_{insert,i},for\;i=1,2,...,N_{chan} \end{array}$$ (2)

which can be solved as a $N_{chan}$ x $N_{chan}$ linear system. For the case of a single channel, this simplifies to $f_{chan,1}=p_{insert,1}/(p_{insert,1}+p_{remove,1})$. We note that for all simulations, the initial seeding probability is set equal to the steady-state fraction, i.e., $p_{seed,i}=f_{chan,i}$, to mitigate initial transient behavior in the model. Further, we define model parameters such that an increase in trafficking rate results in both more frequent channel removal and insertion in a manner that maintains the steady-state fraction $f_{chan}$. Specifically, we define the removal probability $p_{remove}$ as proportional to the channel trafficking rate $r_{traffic}$ (where $p_{remove}=r_{traffic}\delta t$ and δt=1 is a nominal time step), and subsequently $p_{insert}$ is defined from $p_{remove}$ and $f_{chan}$ from Eq (2).

#### Channel-channel interactions and channel diffusion.

If the pixel selected is occupied but not removed, then the channel may move to one of its $N_{move}$ unoccupied neighbors, based on both channel-channel interactions and diffusion. Specifically, the probability of movement to an unoccupied neighboring pixel is determined from a Boltzmann distribution based on the thermodynamic energy differences between the current channel position and the possible unoccupied neighbors. As described further below, movements with negative energy differences are more energetically favorable and thus have a higher probability.

For a channel type *i*, we define the homotypic channel-channel interaction energy $H_{cc}^{ii}$ and heterotypic interaction energy $H_{cc}^{ij}$ for i≠j. Note that it is convenient to define these values as elements in a symmetric matrix, since $H_{cc}^{ij}=H_{cc}^{ji}$. Additionally, we define an energy term $H_{diff,i}$ associated with membrane diffusion that depends on the channel diffusion probability $p_{diff,i}$. We define the relationship between $H_{diff,i}$ and $p_{diff,i}$ below, but clarify here that these have an inverse relationship, i.e., higher diffusion probability is more energetically favorable, and thus smaller $H_{diff,i}$.

We define the energy of the current position as *H*_0_ by summing over all values of $H_{cc}$ for both homotypic and heterotypic channel-channel interactions in the Moore neighborhood of the selected channel. We repeat this process to calculate the energy $H_{m}$ associated with the channel moving to each of the $m=1,2,...,N_{move}$ unoccupied neighbors (i.e., we calculate the energy if the channel moved each of the possible unoccupied positions). From these values, we calculate the energy difference for each *m* possible, $\Delta H_{m,i}=H_{m}+H_{diff,i}-H_{0}$.

Finally, the channel of type *i* moves to unoccupied neighbor *m* with probability given by the Boltzmann distribution:


$$\begin{array}{c} p_{move,m}^{i}=\frac{\exp(-\Delta H_{m,i}/kT)}{\sum_{q=1}^{N_{move}+1}\exp(-\Delta H_{q,i}/kT)}=\frac{\exp(-\Delta H_{m,i}/kT)}{\sum_{q=1}^{N_{move}}\exp(-\Delta H_{q,i}/kT)+1} \end{array}$$ (3)

where *k* is the Boltzmann constant, *T* is the thermodynamic temperature, and we define the probability of the $\left(N_{move}+1\right)$th ‘move’ as the channel not moving, which by definition has no energy difference and thus $\Delta H_{N_{move}+1,i}=0$. Note that we define $H_{cc}$ terms in units of *kT*, such that it is not necessary to introduce an additional value for parameter *T*. The channel either moves to unoccupied neighbor *m* or does not move based on these probabilities.

This complete process is then iterated for a specified number of time points, each time selecting a new random pixel. Time is specified in terms of Monte Carlo Steps (MCS), where one MCS corresponds to $N_{x}N_{y}$ iterations, such that each pixel is selected on average once per MCS.

#### Connection between diffusion model parameters and biophysical measurements.

The energy associated with diffusion $H_{diff,i}$ is related to the channel membrane diffusion probability $p_{diff,i}$ and coefficient $D_{chan,i}$ as follows: First, the probability of a channel moving to one of its unoccupied neighbors solely due to diffusion (and not channel-channel interactions) is defined as $p_{diff,i}=D_{chan,i}\Delta t/4\Delta x^{2}$, where Δt is the time step (in physical units of time) and Δx is the spatial grid size (in physical units of distance). This probability is limited to values ≤1/8, with 1/8 corresponding with a channel guaranteed to move to one of its 8 Moore neighbors. While this probability definition is convenient for a random walk model of pure diffusion, it is not consistent with the channel-channel interaction energy framework.

Thus, we need to define the energy associated with diffusion $H_{diff,i}$ to be consistent with the value of $p_{diff,i}$. Specifically, we define the relationship $p_{diff,i}=\exp(-H_{diff,i})/(8\exp(-H_{diff,i})+1)$ based on the Boltzmann distribution described above, from which we can define $H_{diff,i}=-\ln(p_{diff,i}/(1-8p_{diff,i}))$. For all simulations and channel types, we define $p_{diff,i}=1/9$, which corresponds with $H_{diff,i}=0$, consistent with the channel being equally likely to move to one of its 8 neighbors or not move, which also conveniently defines the energy associated with channels interacting with free membrane as 0, such that negative (positive) $H_{cc}$ describes channel-channel interactions more (less) energetically favorable than free membrane. Note that for this study, we present results in normalized time and distance (i.e., in MCS and pixels, respectively), but the modeling framework can be naturally generalized based on physical channel dimensions and diffusion coefficients.

Thus, collectively, the parameters for channel insertion, removal, and diffusion probabilities and channel-channel interaction terms define the model.

### Model analysis

Unless otherwise specified, simulations were run for 10,000 MCS. Clusters were defined as groups of at least 3 channels, sharing adjacent pixel connections, utilizing the binary connected component (bwconncomp) function in the Image Processing Toolbox in MATLAB. Cluster mean and standard deviation measurements were calculated by taking the mean over the final 5,000 MCS. For simulations with two channel types, the nearest neighbor distribution between channel type 1 and 2, is calculated by the pairwise distance (pdist2 function in MATLAB) between all possible combinations of channels of type 1 and 2.

Simulation code is provided as S1 Code.

## Results

### Dynamics of single channel type clustering

**Fig 1:**
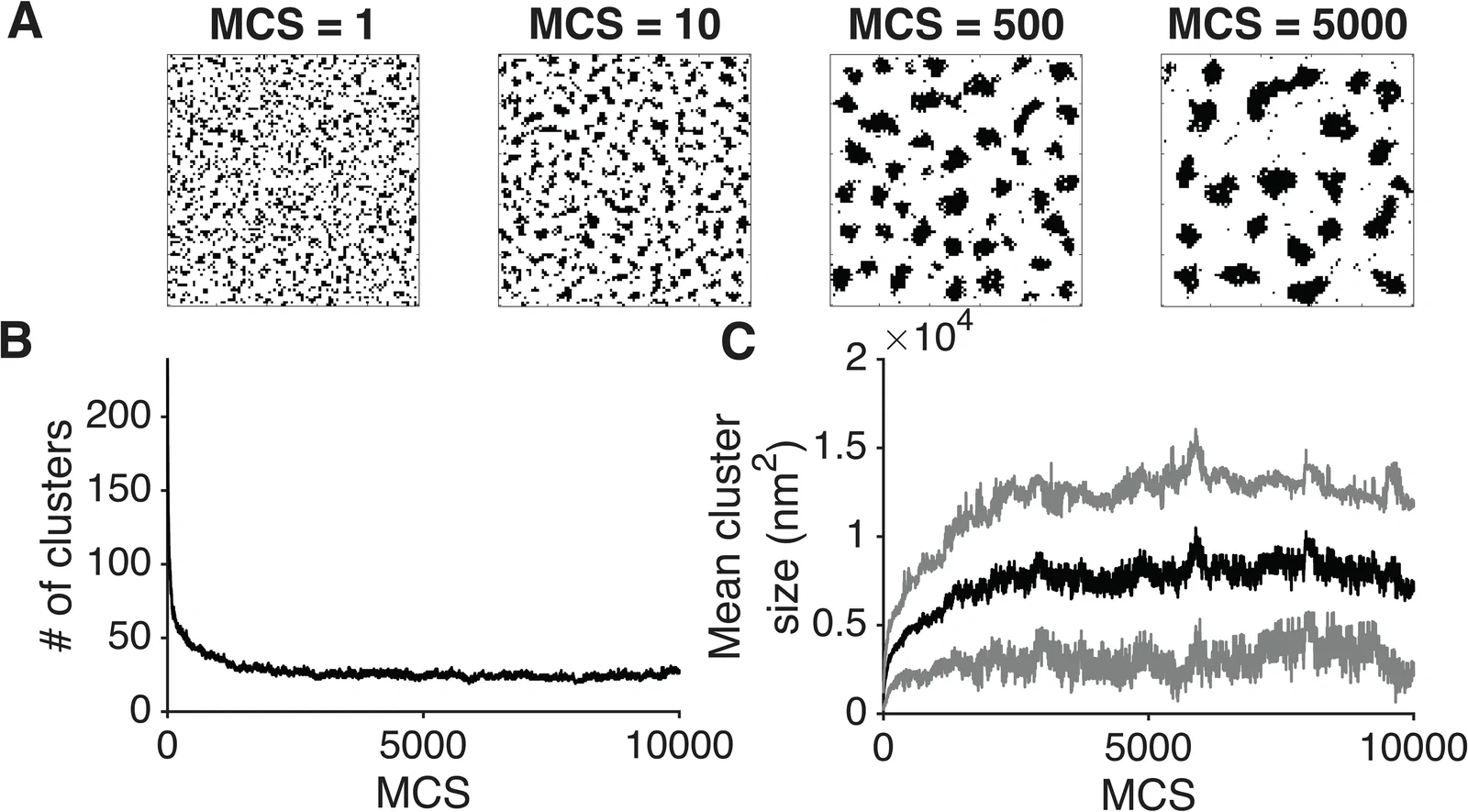
Temporal evolution of single channel type clustering. **(A)** Spatial distribution of a single channel type on a 100 x 100 (1 μm x 1 μm) membrane grid shown at four time points (1, 10, 500, and 5000 Monte Carlo steps [MCS]). **(B)** The total number of clusters and (C) mean cluster size (black), with +/- 1 standard deviation (grey) are shown as functions of time. The simulation demonstrates the progression from an initially random distribution to the formation of small clusters, followed by cluster growth and eventual establishment of a dynamic steady state. Parameters: channel fraction ($f_{chan}$) = 0.2, trafficking rate ($r_{traffic}$) = 0.0005, and homotypic interaction energy ($H_{cc}$) = -1.

We first modeled the minimal system containing a single channel type ($N_{chan}$ = 1) to investigate ion channel dynamics on the cell membrane for parameters that result in robust clustering (Fig 1A, S1 Video). After initial random seeding (MCS = 1), small clusters began to form through diffusion-mediated aggregation (MCS = 10). By intermediate time points (MCS = 500), more substantial clustering emerged with significant variability in cluster size. In the later stages (MCS = 5000), the system approached a steady state characterized by a stable distribution of cluster sizes, though individual clusters continued to undergo dynamic remodeling through trafficking events and diffusion. Throughout this temporal evolution, the Monte Carlo approach generated variability in cluster morphology and spatial distribution, reflecting the inherent randomness of clustering processes at the cell membrane. The total number of clusters initially decreases, while mean cluster size increases, in time, reflecting initial cluster formation, with both measures reaching steady-states with small fluctuations (Fig 1B, 1C).

**Fig 2:**
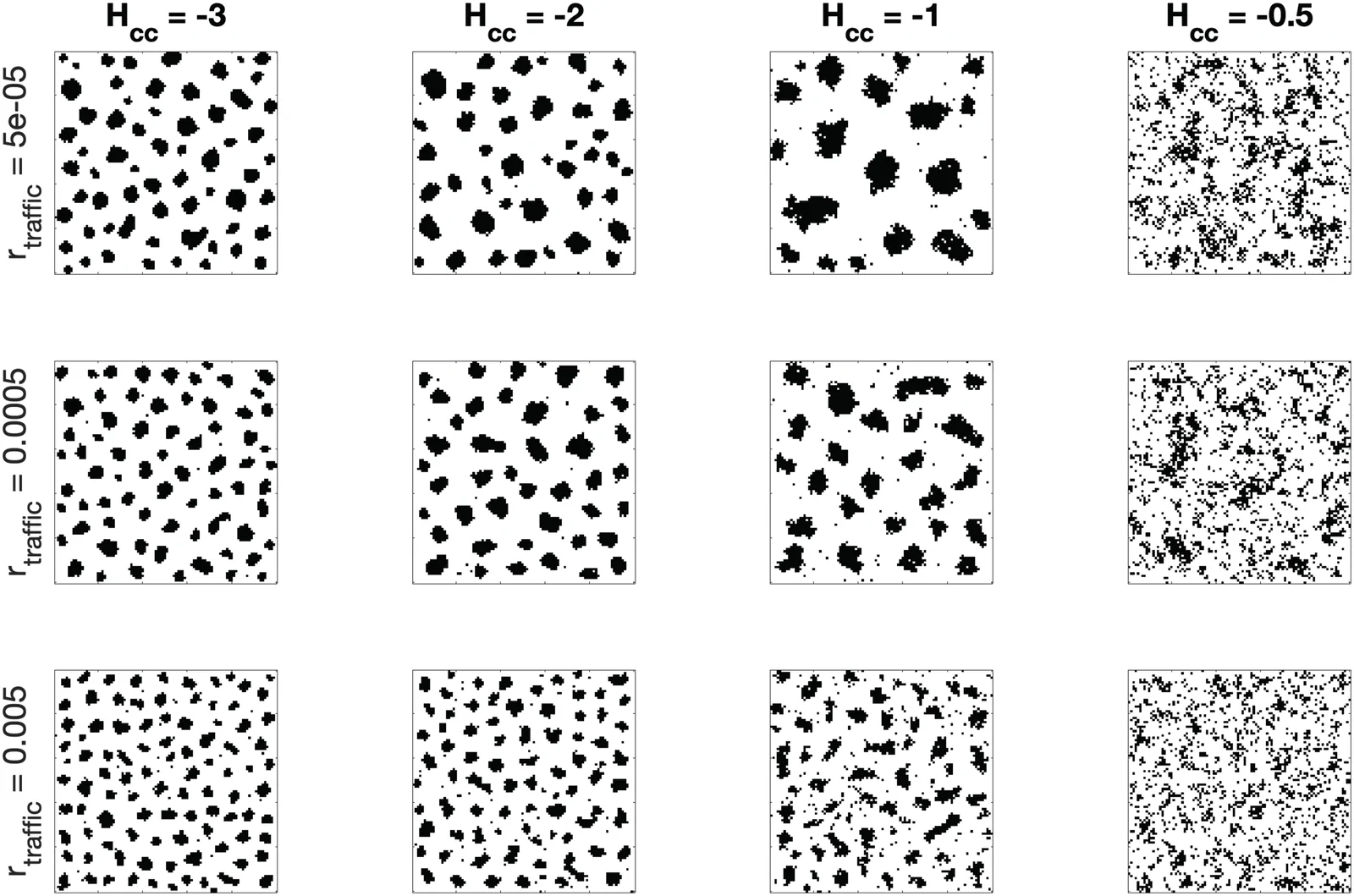
Effects of trafficking rate and interaction energy on single channel clustering. Clusters for a single channel type with varying trafficking rates ($r_{traffic}$, rows) and homotypic interaction energies ($H_{cc}$, columns). Channel fraction ($f_{chan}$) = 0.2. Lower trafficking rates consistently promote larger clusters, while the optimum clustering occurs at $H_{cc}=-1$, with reduced cluster sizes at both more and less negative interaction energies.

The effects of varying trafficking rates and interaction energies on channel clustering are illustrated in Fig 2. As the trafficking rate ($r_{traffic}$) decreased, more stable and larger clusters formed due to the reduced turnover of channels. Interestingly, the relationship between interaction energy ($H_{cc}$) and cluster size was non-monotonic, with the largest clusters observed at $H_{cc}=-1$, such that the largest and most stable channel clusters when combining slow trafficking rates and intermediate interaction energy of $H_{cc}=-1$.

**Fig 3:**
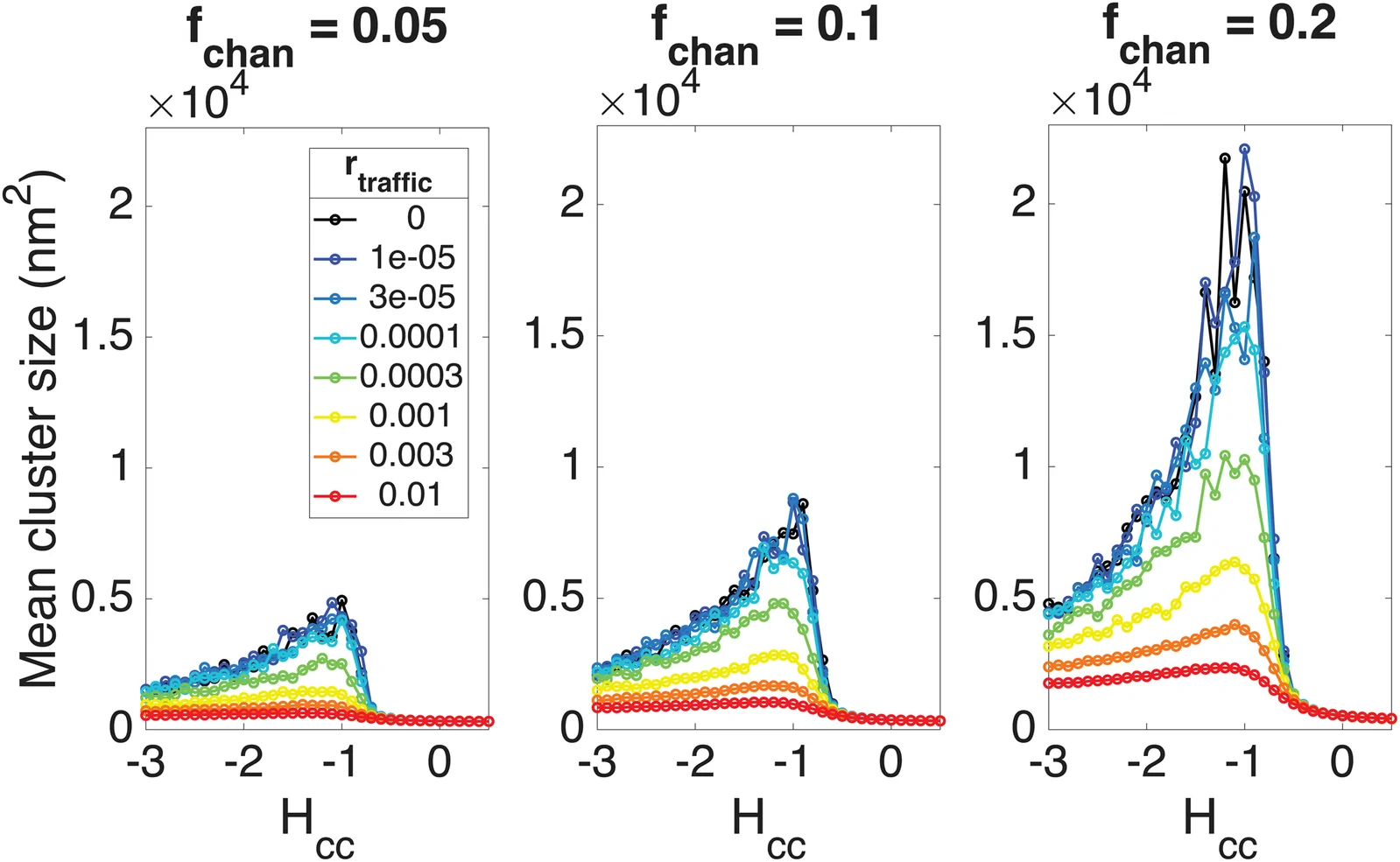
Quantitative analysis of clustering parameters for a single channel type. Mean cluster size is shown as a function of channel fraction ($f_{chan}$), trafficking rate ($r_{traffic}$), and homotypic interaction energy ($H_{cc}$).

To quantitatively assess how these parameters affect clustering behavior, we systematically varied channel fraction ($f_{chan}$), trafficking rate ($r_{traffic}$), and homotypic interaction energy ($H_{cc}$) (Fig 3). Notably, the mean cluster size exhibited a biphasic relationship with interaction energy, with a peak occurring near $H_{cc}=-1$. This non-monotonic behavior suggests that while moderately negative interaction energies are necessary to promote clustering, excessively strong interactions constrain channel mobility and limit cluster growth. As shown in Fig 2, increased trafficking rates reduce cluster size. Additionally, higher channel fractions, such that there are more channels on the membrane, also generally increased cluster size. These relationships highlight the complex interplay between channel density, interaction strength, and membrane turnover in determining the steady-state organization of membrane channels.

### Two-channel system: Competition between homotypic and heterotypic interactions

**Fig 4:**
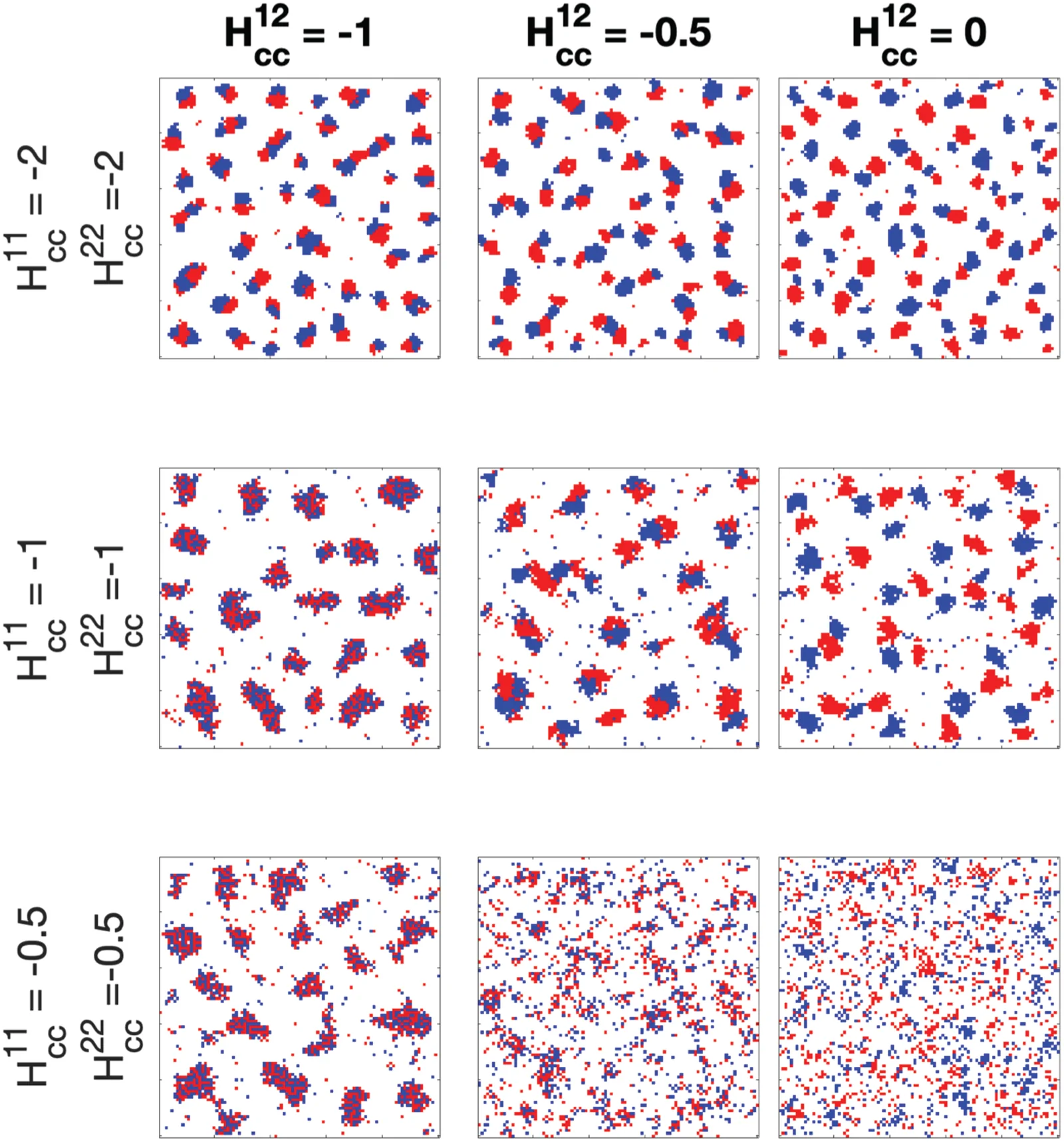
Two-channel clustering with equal homotypic interaction energies. Spatial distributions of two channel types (type 1 in red, type 2 in blue) with equal channel fractions ($f_{chan}=0.1$) and trafficking rates ($r_{traffic}=0.0005$). Each panel represents a different combination of homotypic ($H_{cc}^{11}=H_{cc}^{22}$, rows) and heterotypic ($H_{cc}^{12}$, columns) interaction energies. More negative heterotypic energies promote mixed clustering, while more positive values lead to channel segregation.

We next considered a membrane with two distinct channel types ($N_{chan}=2$), incorporating both homotypic ($H_{cc}^{11}$, $H_{cc}^{22}$) and heterotypic ($H_{cc}^{12}$) interactions. Fig 4 shows the spatial organization of two channel types (red for type 1, blue for type 2) under conditions where both channel types had equal fractions and trafficking rates, while the interaction energies were systematically varied. When homotypic interactions were equivalent for both channel types ($H_{cc}^{11}=H_{cc}^{22}$), the spatial patterns were primarily determined by the heterotypic interaction energy ($H_{cc}^{12}$). For stronger homotypic interaction energy ($H_{cc}^{11}=H_{cc}^{22}=-2$), heterotypic interactions result in a spatial organization of either adjacent or more segregated clusters (S2 Video). For weaker homotypic interaction energies ($H_{cc}^{11}=H_{cc}^{22}=-1$ or -0.5), more negative heterotypic energies promoted the formation of mixed clusters containing both channel types (S3 Video), while more positive heterotypic energies led to segregation of the two channel populations into distinct clusters.

**Fig 5:**
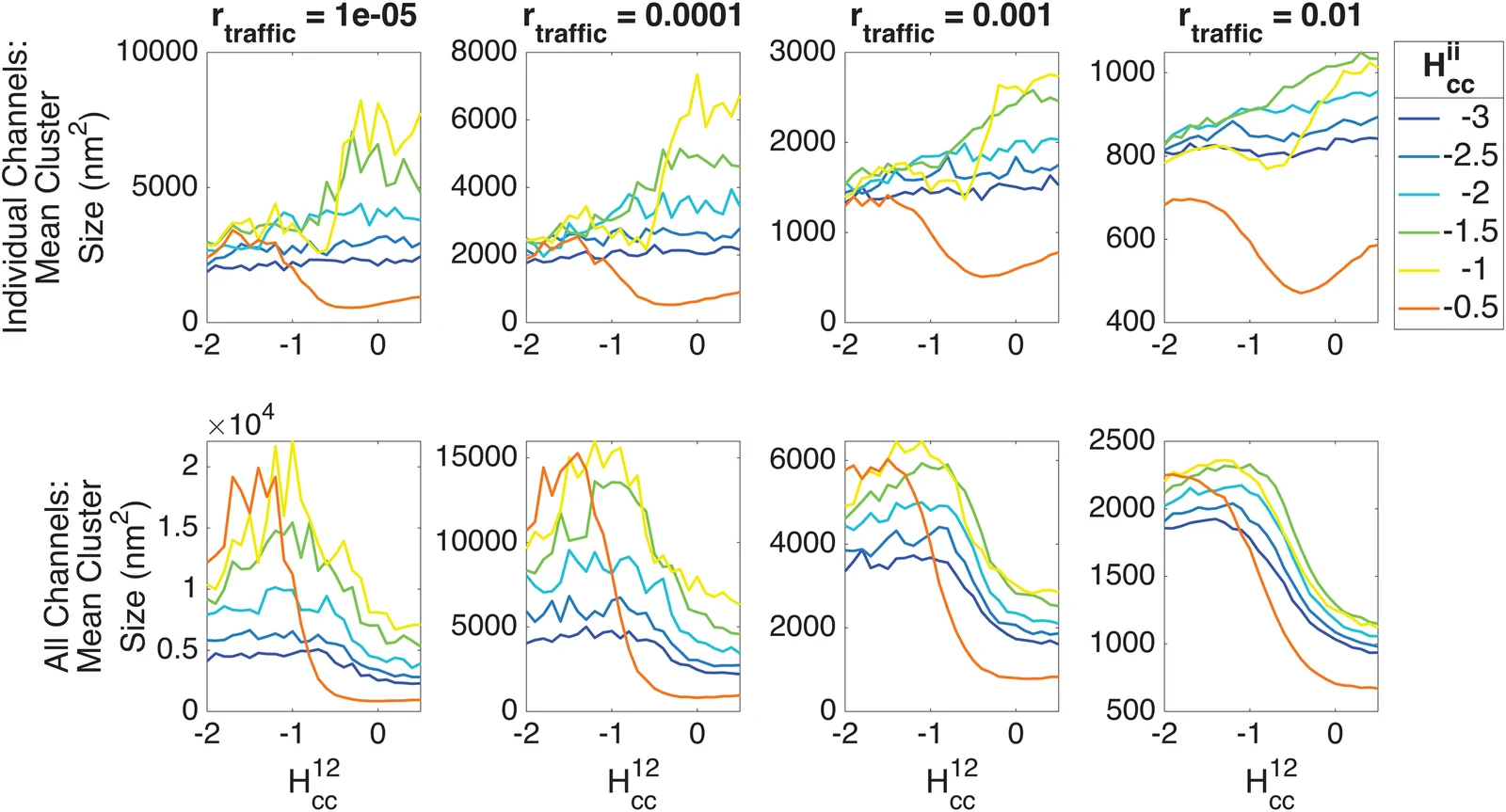
Two-channel clustering with varying interaction energies. Mean cluster size as a function of heterotypic interaction energy ($H_{cc}^{12}$) for different homotypic interaction energies ($H_{cc}^{11}=H_{cc}^{22}=H_{cc}^{ii}$, different lines). Top row: Mean cluster size for clusters containing only individual channel types. Bottom row: Mean cluster size for clusters containing any channel type. Channel fraction $f_{chan}$ for both types was fixed at 0.1.

We next quantify the relationship between interaction energies and clustering size for two channel types with equal homotypic interaction energies (Fig 5). The top panels show the mean cluster size for the individual channel types (averages of both channel type, as the two types have identical properties), while the bottom panels display the mean size of clusters containing all channel types. Consistent with the single channel simulations, the largest cluster sizes occur for homotypic interaction energies near -1 (yellow lines), and similarly, slower trafficking results in larger clusters. As the heterotypic interaction energy ($H_{cc}^{12}$) transitioned from positive to negative values, corresponding with the increase in mixed cluster formation, there is both a decrease in individual cluster size and an increase in total cluster size, particularly when homotypic interactions were also favorable (negative $H_{cc}^{11}$ and $H_{cc}^{22}$ values near -1). Interestingly, this transition point from segregated to mixed clustering generally occurs for heterotypic interaction energies near $H_{cc}^{12}=-0.5$, i.e., half as strong as the optimal homotypic interaction energy, although this transition is more gradual and appears to be influenced by the relative strengths of homotypic versus heterotypic interactions.

**Fig 6:**
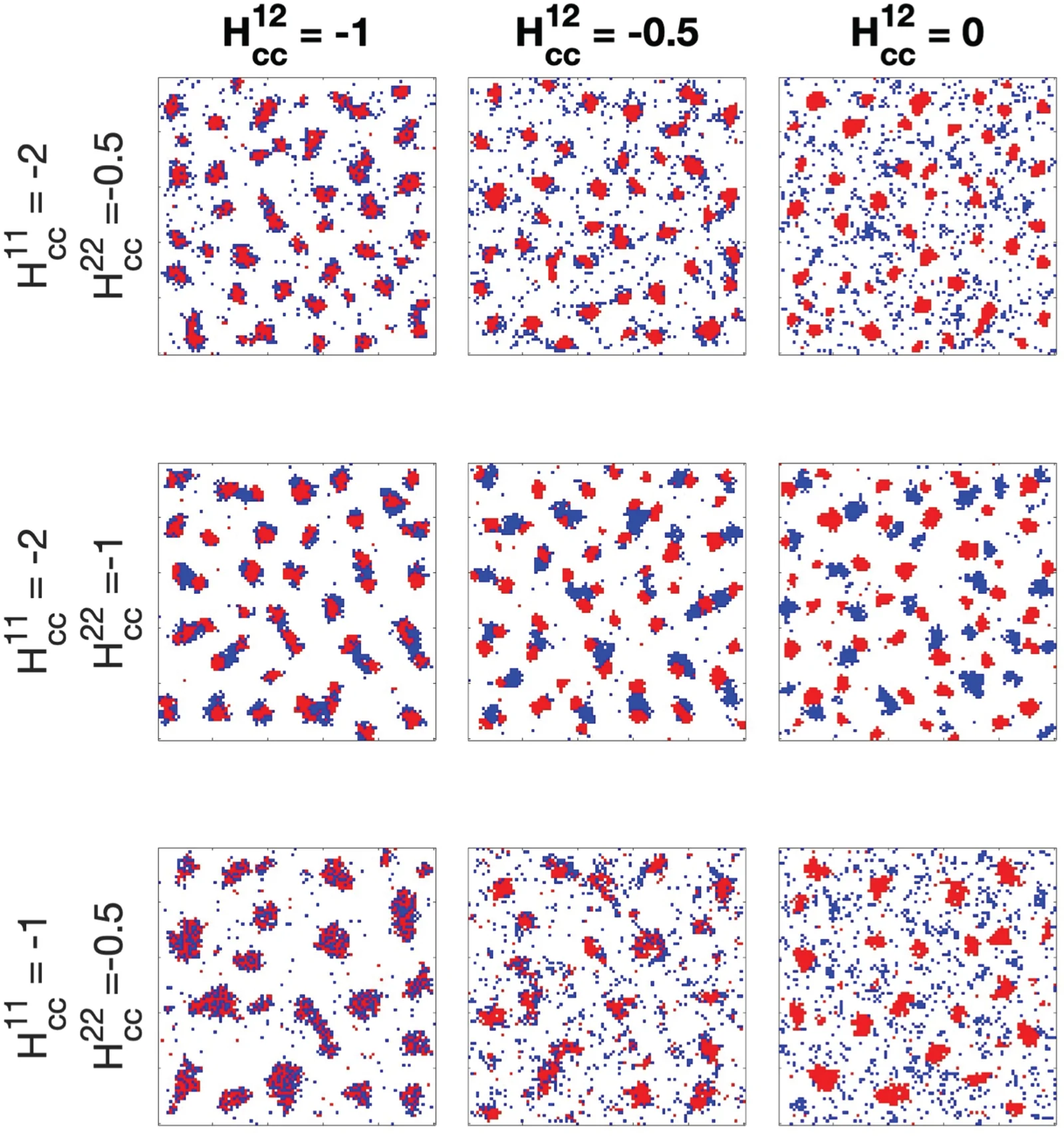
Two-channel clustering with unequal homotypic interaction energies. Spatial distributions of two channel types (type 1 in red, type 2 in blue) with unequal homotypic interaction energies ($H_{cc}^{11}<H_{cc}^{22}$). Channel fractions ($f_{chan}=0.1$) and trafficking rates ($r_{traffic}=0.0005$) were equal for both channel types. Each panel shows a different combination of homotypic and heterotypic interaction energies, revealing asymmetric clustering behaviors for conditions in which the channel types have different homotypic interactions.

We next illustrate the possible complexity of channel organization for cases in which the homotypic interactions differ between channel types (Fig 6). We find that for conditions in which one channel type exhibited stronger homotypic interactions than the other ($H_{cc}^{11}<H_{cc}^{22}$), the channel type with stronger homotypic interactions tended to form more robust clusters, while the heterotypic interaction energy determined whether the second channel type would integrate into these clusters or form separate assemblies (S4 Video). This asymmetry in clustering behavior suggests potential mechanisms for selective recruitment or exclusion of specific channel types within membrane domains.

### Spatial relationships between different channel types

**Fig 7:**
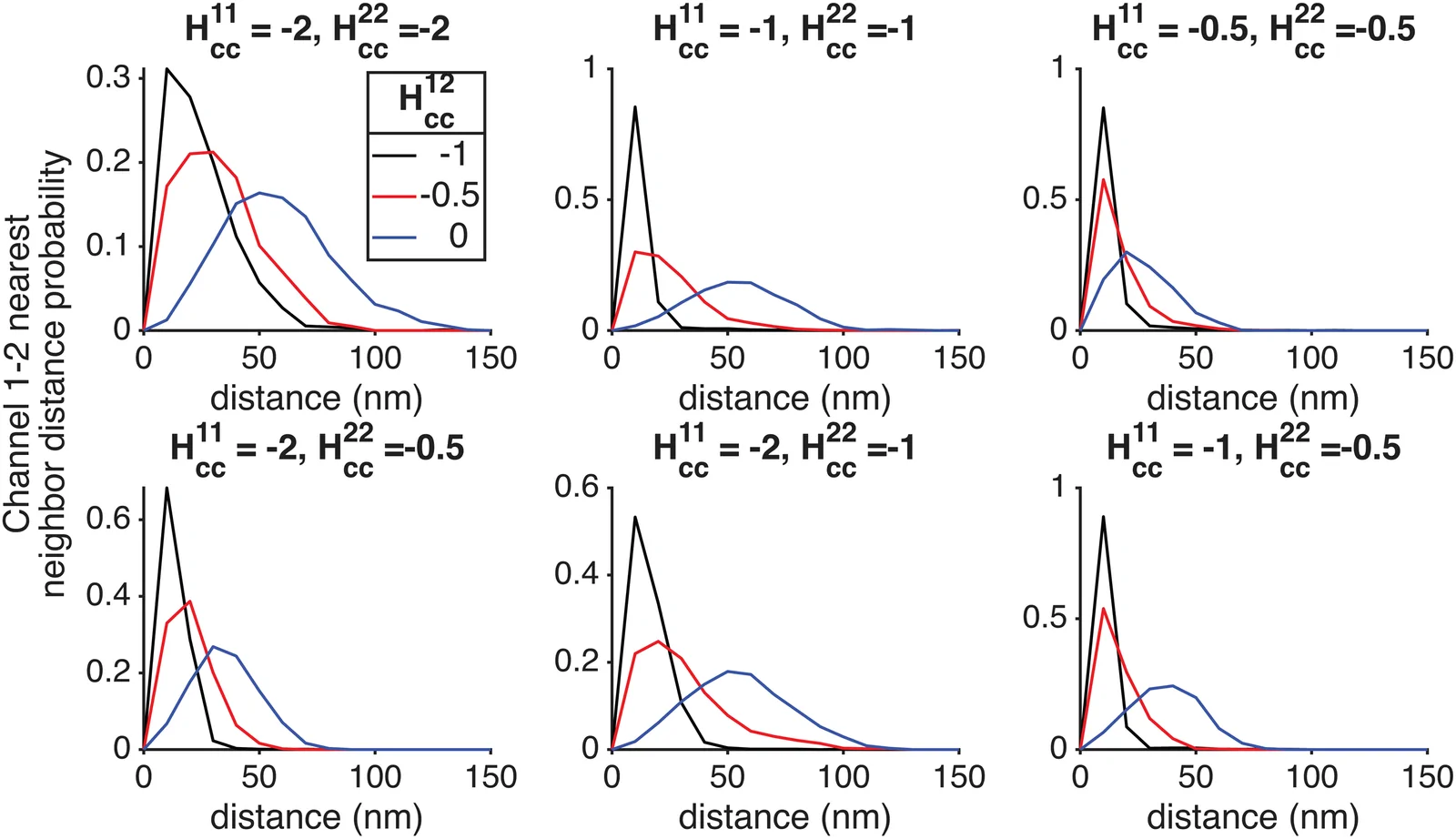
Nearest neighbor distributions between different channel types. Distribution of distances from each type 1 channel to its nearest type 2 channel neighbor for the 18 simulation conditions shown in Figs 4 and 6. Curves shifted toward shorter distances indicate closer association between different channel types (more negative $H_{cc}^{12}$), while curves shifted toward larger distances indicate segregation (more positive $H_{cc}^{12}$).

We further characterize the spatial relationships between different channel types by analyzing the nearest neighbor distributions between type 1 and type 2 channels for the 18 simulations shown in Figs 4 and 6, quantifying channel organization. The nearest neighbor distributions varied considerably depending on the interaction energy parameters (Fig 7). For more energetically favorable heterotypic interactions (more negative $H_{cc}^{12}$), the distributions showed peaks at shorter distances, indicating close association between different channel types. Conversely, unfavorable heterotypic interactions (more positive $H_{cc}^{12}$) resulted in distributions shifted toward larger distances, indicating spatial segregation. Interestingly, for both channel types having the same homotypic interactions (top row), stronger homotypic interactions generally resulted in wider distributions, also indicating greater separation between channel types. In contrast, asymmetric homotypic interactions (bottom row) resulted in a more complex relationship.

### Emergent properties in three-channel systems

**Fig 8:**
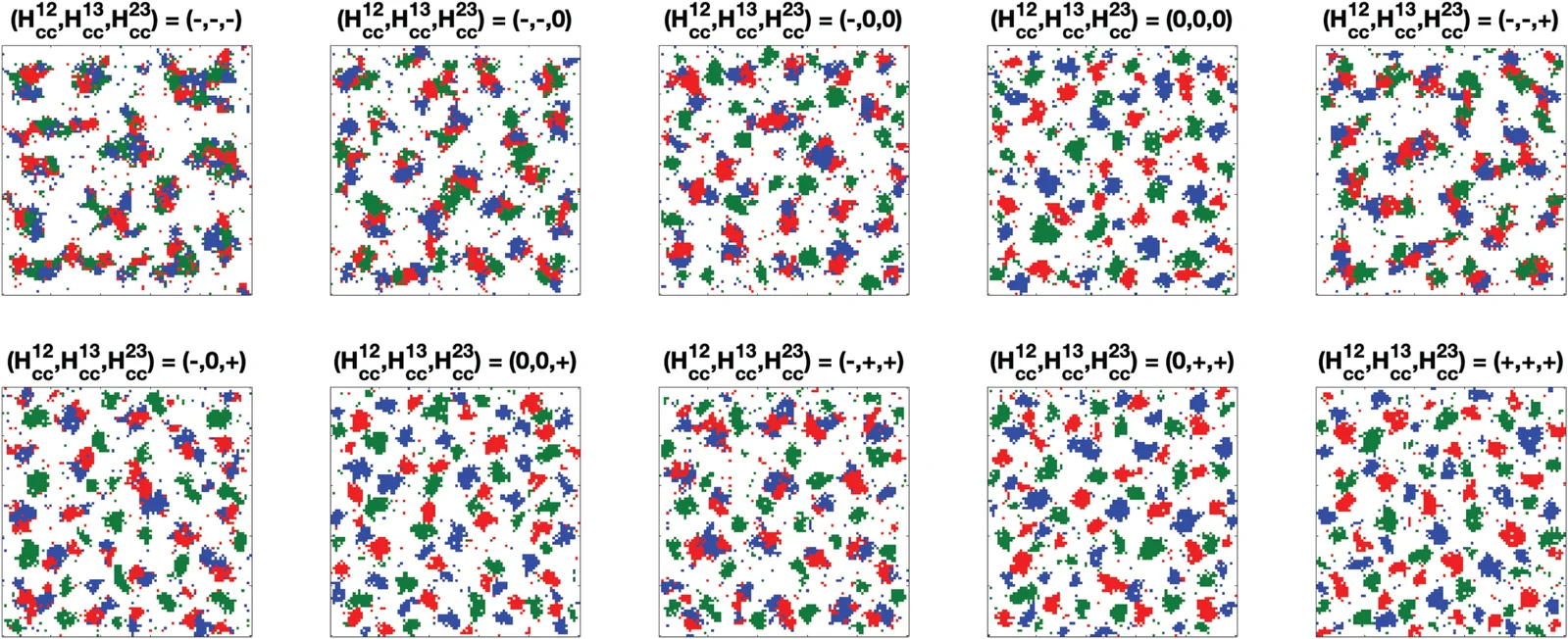
Emergent properties in three-channel systems. Simulations with three channel types (type 1 in red, type 2 in blue, type 3 in green) with equal channel fractions ($f_{chan}=0.1$) and trafficking rates ($r_{traffic}=0.0005$) and homotypic interaction energies ($H_{cc}^{11}=H_{cc}^{22}=H_{cc}^{33}=-1$). Heterotypic interaction energies vary across panels, where “-” indicates negative energy (-0.5), “0” indicates zero energy, and “+” indicates positive energy (+0.5). The resulting patterns demonstrate complex organizational principles emerging from the interaction network among the three channel types.

Finally, we generalize the system to the case of three interacting channel types ($N_{chan}=3$). We note that the combinatorial possibilities for interactions increase substantially with three (or more) channels, in addition to variations in trafficking rates and channel densities, and thus we aim to illustrate a small subset of possible channel properties. We vary the heterotypic interaction energies for three channel types, all with equivalent channel fractions and strong homotypic interaction energies (Fig 8). The resulting patterns reveal complex emergent spatial patterns. Particularly noteworthy are conditions where certain pairs of channel types exhibit favorable interactions while others do not, leading to distinct clustering patterns. These complex arrangements demonstrate how the selective affinity between different channel types could potentially organize the membrane into functionally specialized regions.

**Fig 9:**
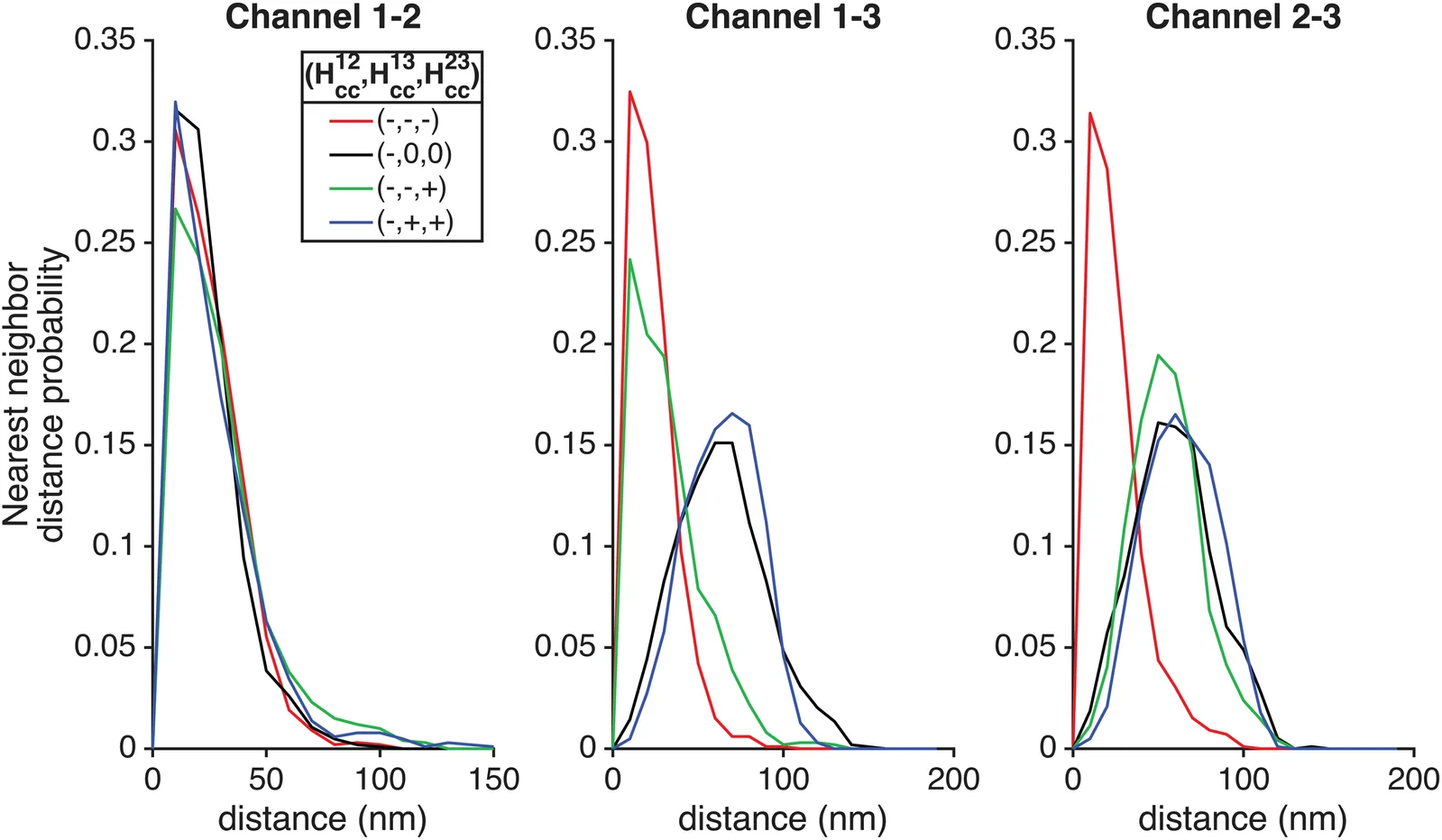
Nearest neighbor distributions in three-channel systems. Distribution of nearest neighbor distances for each channel type pair, for a subset of the simulation conditions shown in Fig 8. All three channel types have equal channel fractions ($f_{chan}=0.1$) and trafficking rates ($r_{traffic}=0.0005$) and homotypic interaction energies ($H_{cc}^{11}=H_{cc}^{22}=H_{cc}^{33}=-1$). Heterotypic interaction energies vary, where “-” indicates negative energy (-0.5), “0” indicates zero energy, and “+” indicates positive energy (+0.5).

We analyzed the nearest neighbor distributions for a subset of these simulations to quantify how the interactions between all channel types influence the overall spatial distribution, and critically, how the interactions between one pair of channel types influences a different pair (Fig 9). With channel types 1 and 2 maintaining energetically favorable interactions (negative $H_{cc}^{12}$), we varied the interactions between channel types 1 and 3 and types 2 and 3: both energetically favorable (negative $H_{cc}^{13}$ and $H_{cc}^{23}$, red), unfavorable (positive $H_{cc}^{13}$ and $H_{cc}^{23}$, blue), neutral (negative $H_{cc}^{13}=H_{cc}^{23}=0$, black), and one favorable and one unfavorable (negative $H_{cc}^{13}$, positive $H_{cc}^{23}$, green).

We find that differences in the channel type 1–3 and 2–3 interactions modulate the nearest neighbor distribution for channel type 1–2 (Fig 9, left). Interestingly, channel types 1 and 2 exhibit the closest spatial association for neutral 1–3 and 2–3 interactions (black; mean nearest neighbor distance of 2.397 pixels). The next closest channel type 1 and 2 association occurs for both favorable 1–3 and 2–3 interactions (red; mean of 2.503 pixels), followed by both unfavorable (blue; mean of 2.757 pixels). Asymmetric channel type 1–3 and 2–3 interactions produce the largest channel type 1 and 2 separation (green; mean of 2.953 pixels), with a reduced peak at shorter distances and extended tail for longer distances.

The nearest neighbor distributions for channel type 1 and 3 (Fig 9, middle) and type 2 and 3 (Fig 9, right) also reflect competing interactions effects. Notably, for both favorable channel type 1–2 and 1–3 interactions, channel type 2–3 interactions modulate both distributions. Unfavorable channel type 2-3 interactions (green) result in an expected shift toward larger channel type 2–3 separation (right), relative to favorable channel type 2–3 interactions (red). However, the unfavorable channel type 2–3 interactions also result in greater spatial channel type 1–3 separation (middle), with a reduced peak at shorter distances (green).

## Discussion

In this study, we have developed a stochastic computational model to investigate the principles governing ion channel organization on cell membranes. By systematically varying key parameters such as channel density, trafficking rates, and interaction energies, we have uncovered several fundamental properties of channel clustering that provide insight into the mechanisms underlying membrane domain formation.

One of the most striking findings from our single-channel simulations is the biphasic relationship between interaction energy and cluster size, with optimal clustering occurring at intermediate interaction strengths ($H_{cc}$ near -1). This non-monotonic behavior suggests that while attractive forces are necessary for cluster formation, excessively strong interactions rather impede the growth of large clusters by creating “traps” that restrict channel mobility. Our computational results provide a theoretical framework to explain such counterintuitive observations. The temporal dynamics also illustrate the importance of considering membrane organization as a non-equilibrium process. The progression from random distribution to steady-state clustering involves multiple phases, emphasizing that cellular regulation of trafficking rates could serve as a mechanism to control not only the extent but also the rate of cluster formation. This aligns with recent live-cell imaging studies demonstrating that neurons can rapidly modulate channel clustering in response to activity changes through regulated trafficking [35].

Notably, a version of our model with a single channel and no turnover ($r_{traffic}=0$) is equivalent to the Ising model with conserved spin [36]: a spin of 1 can be considered analogous to a channel, while a spin of -1 is empty membrane. Further, while in our model the temperature *T* is kept constant, varying $H_{cc}$ is equivalent to varying the ratio $T/T_{c}$ in the Ising model. Therefore, in Figs 1-3 above, we observe a similar phase transition as $H_{cc}$ increases. Moreover, we observe the same tendency of the intermediary clusters to merge and ultimately form a single large cluster. The high values of $H_{cc}$ are therefore equivalent to a low $T/T_{c}$ ratio, and the same long lag time of cluster formation as described in reference [36] is replicated. However, we note that the addition of a biologically realistic channel turnover or multiple channel species breaks the equivalence to the conserved spin Ising model: turnover suppresses the drive to form a single cluster, setting a finite characteristic cluster size dependent on the turnover rate; multiple channels lead to complex emergent structures, as detailed in Figs 4-8.

Investigation of two-channel systems revealed how the interplay between homotypic and heterotypic interactions creates diverse spatial patterns. When homotypic interactions were equal between channel types, heterotypic interaction energy primarily determined whether channels formed mixed or segregated clusters. However, in the more biologically relevant scenario where homotypic interactions differed between channel types, we observed asymmetric clustering behaviors that suggest potential mechanisms for selective recruitment or exclusion of specific channel types within membrane domains. These findings extend previous work observing preferential co-clustering of certain channel subtypes, by providing a mechanistic explanation for how such selective co-clustering might arise from the balance of interaction energies [26]. Nearest neighbor analysis provided quantitative metrics for characterizing spatial relationships between different channel types. Future work could integrate and fit experimental super-resolution imaging data, thus enabling predictions of interaction parameters from observed channel distribution observations [27].

Our results also parallel studies of membrane lipid composition. Membrane lipids (phospholipids and cholesterol) can have attractive or repulsive contact interactions, depending on their chemical structure. In a modified Ising model (for a current review see [37]), it has been shown that two- or three species of lipids form distinct lipid domains or phases, with a particular local density of each species depending on the modeled species and their interactions.

Simulations of three-channel systems revealed interesting emergent organizational principles. The hierarchical clustering patterns and specialized domain formation observed under certain interaction conditions suggest how the selective affinity between different channel types could organize the membrane into functionally specialized regions. These findings may explain the complex distribution of channel and structural proteins observed in specialized neuronal compartments such as the axon initial segment [24].

The channel clustering model builds upon several modeling frameworks, including random walk, the Ising model, Cellular Potts, and cellular automata models, all lattice-based models simulated with Monte Carlo methods that consider probabilistic movement of ‘particles’, ‘cells’, etc. Sato and colleagues previously modeled channel trafficking and cluster growth, fitting trafficking parameters to either constant or time-dependent values based on experiments [25]. However, this study did not consider channel-channel interactions or channel diffusion, nor the presence of other channel types (and potential interactions). Goldman et al modeled protein-protein interactions, but considered neither trafficking nor diffusion [38].

Recent studies have sought to bridge the relation between ion channel cluster and their function. There is growing evidence that various ion channels gate cooperatively, with important implication for cardiac arrhythmias or epilepsy [39]. The organization of ryanodine receptors (RyR) in clusters, in rat ventricular myocytes) is critically important for synchronized calcium releases that drive cell contraction. Baddeley and colleagues have shown that a simple cluster growth model (i.e., simple addition/removal without diffusion) explains the size distribution of clusters as obtained through optical single-channel resolution imaging [40]. Further, Suma and colleagues show, in an Ising-like model, that including diffusing lipids and K+ channels, that channels can form clusters. Crucially, they include a representation of K+ channel gating that depends on local interactions with neighboring lipids or channels [41]. Thus, they show that the channels exhibit complex cooperativity as well as long term memory effects, demonstrating that their behavior is directly influenced by local membrane structure. Building upon these studies, a natural continuation of our present work would be to link cooperative channel gating and electrophysiological models with the channel clustering model described here. This would allow us to go beyond representations of a single type of channel, and describe the behavior of potentially complete sets of ion channels distributed in complex membrane domains determined by pairwise channel interactions.

Further, we note that our framework is generalizable to represent a wide range of channel biophysical properties, including a range of spatial and temporal scales. Importantly, key model parameters can be directly estimated from biophysical measurements of these scales. Estimates of the diffusion coefficient on the order of 0.05 μm^2^/sec [42] and the pixel size Δx of 10 nm, with the specified diffusion probability $p_{diff}$, set the physical time step Δt of 1 msec. Thus, the nominal $r_{traffic}$ rate of 0.0005 per MCS sets the time scale for trafficking at 20,000 sec or approximately 5.6 hours, accounting for $N_{x}N_{y}$ time steps per MCS, which is consistent with recent work noting the highly dynamic nature of ion channel trafficking on the order of minutes to hours [43]. Importantly, model parameters considered in our study are consistent with the range of physiological measurements, and further that a wide range of diffusion and trafficking spatiotemporal scales from specific channels can be readily accounted for by model parameter variation.

We note several limitations of our current model. First, the lattice-based approach, while computationally efficient, imposes geometric constraints that may not fully capture the fluidity of biological membranes. Additionally, our model does not explicitly incorporate membrane lipids or cytoskeletal elements, which are known to influence channel distribution. Finally, the interaction energies are treated as fixed parameters, whereas in biological systems these interactions may be dynamically regulated, e.g., through post-translational modifications or conformational changes, both in space and time. Future extensions of this work could address these limitations by incorporating lipid domains, cytoskeletal interactions, and activity-dependent regulation of channel properties. Additionally, expanding the model to include spatial and/or temporal heterogeneity in trafficking rates could provide insight into how cells establish and maintain polarized channel distributions. Integration with electrophysiological models would also allow investigation of how different clustering patterns influence cellular excitability and signal processing, in particular in the context of dynamic nanodomains [44]. Another promising direction would be to develop methods for directly estimating interaction energies from experimental data, potentially through statistical and/or machine learning-based approaches that compare simulated and observed channel distributions. Such parameter estimation would facilitate the application of our model to specific channel types and subcellular settings, facilitating direct testing of model predictions.

## Conclusion

Our computational framework provides novel insights into the principles governing ion channel organization and generates testable predictions about how channel density, trafficking dynamics, and interaction energies collectively shape membrane domain formation. The non-intuitive relationships uncovered between these parameters highlight the importance of computational approaches for understanding complex biological systems where multiple processes operate simultaneously across different spatial and temporal scales. Future integration of this modeling approach with experimental measurements promises to advance our understanding of how membrane organization contributes to cellular function in both health and disease.

## Supporting information

S1 VideoTemporal evolution of single channel type clustering.The progression from an initially random distribution to the formation of small clusters, followed by cluster growth and eventual establishment of a dynamic steady state. Parameters: channel fraction ($f_{chan}$) = 0.2, trafficking rate ($r_{traffic}$) = 0.0005, and homotypic interaction energy ($H_{cc}$) = -1. Data shown in Fig 1. Filename: movie_single_chan.mp4.(MP4)

S2 VideoTwo-channel clustering with equal strong homotypic and intermediate heterotypic interaction energies.Spatial distributions of two channel types (type 1 in red, type 2 in blue), with equal channel fractions ($f_{chan}=0.1$), trafficking rates ($r_{traffic}=0.0005$), and homotypic ($H_{cc}^{11}=H_{cc}^{22}=-2$) and heterotypic ($H_{cc}^{12}=-1$) interaction energies. Data shown in Fig 4 (top row, left column). *Filename: movie_hom_two_chan_Hcc-2.mp4*.(MP4)

S3 VideoTwo-channel clustering with equal intermediate homotypic and intermediate heterotypic interaction energies.Spatial distributions of two channel types (type 1 in red, type 2 in blue), with equal channel fractions ($f_{chan}=0.1$), trafficking rates ($r_{traffic}=0.0005$), and homotypic ($H_{cc}^{11}=H_{cc}^{22}=-1$) and heterotypic ($H_{cc}^{12}=-1$) interaction energies. Data shown in Fig 4 (middle row, left column). *Filename: movie_hom_two_chan_Hcc-1.mp4.*(MP4)

S4 VideoTwo-channel clustering with asymmetric homotypic and intermediate heterotypic interaction energies.Spatial distributions of two channel types (type 1 in red, type 2 in blue), with equal channel fractions ($f_{chan}=0.1$), trafficking rates ($r_{traffic}=0.0005$), and homotypic ($H_{cc}^{11}=-2,\;H_{cc}^{22}=-0.5$) and heterotypic ($H_{cc}^{12}=-1$) interaction energies. Data shown in Fig 6 (top row, left column). *Filename: movie_het_two_chan_Hcc_mixed.mp4.*(MP4)

S1 Code MATLAB simulation code. MATLAB simulation code that performs channel clustering simulations.
(M)
